# Optimization of instant sweet osmanthus white tea: formulation, sensory evaluation, and antioxidant properties

**DOI:** 10.3389/fnut.2025.1538848

**Published:** 2025-02-19

**Authors:** Yuhua Yang, Li Fan, Yiqin Lin, Yan Huang, Jianming Zhang, Shaohua Li, Chunhua Ma, Xi Cheng, Wee Yin Koh, Thuan-Chew Tan

**Affiliations:** ^1^College of Tea and Food Science, Wuyi University, Wuyishan, China; ^2^Food Technology Division, School of Industrial Technology, Universiti Sains Malaysia (USM), Penang, Malaysia; ^3^Faculty of Food Science and Nutrition, Universiti Malaysia Sabah, Kota Kinabalu, Sabah, Malaysia; ^4^Renewable Biomass Transformation Cluster, School of Industrial Technology, Universiti Sains Malaysia (USM), Penang, Malaysia

**Keywords:** sweet osmanthus, white tea, functional beverage, orthogonal test, product development, *in vitro* antioxidant

## Abstract

**Objective:**

This study aims to optimize the formulation of instant sweet osmanthus white tea (IOWT) and evaluate its antioxidant activities through *in vitro* assays.

**Methods:**

Single-factor and orthogonal experiments were conducted to investigate the impacts of sweet osmanthus-to-white tea ratio and the concentrations of *β*-cyclodextrin, erythritol, and citric acid on sensory properties and total flavonoids content (TFC) of IOWT. Mixtures of the dried ingredients were spray-dried to produce IOWT.

**Results and discussion:**

The optimal formulation of IOWT was as follows: sweet osmanthus-towhite tea ratio of 1:5, 4% *β*-cyclodextrin, 6% erythritol, and 0.5% citric acid. This optimized IOWT formulation obtained the highest sensory score of 89.5 and a TFC of 25.76%. Using ascorbic acid as a positive control, the *in vitro* antioxidant activities of the optimized IOWT formulation were assessed by measuring its ability to scavenge DPPH radicals, hydroxyl radicals, superoxide anion radicals, and ABTS radicals. At a concentration of 1.0 mg/mL, the optimized IOWT formulation exhibited scavenging rates of 88.01, 94.99, 97.57, and 99.11% against DPPH, hydroxyl radicals, superoxide anion radicals, and ABTS radicals, respectively, indicating strong *in vitro* antioxidant activities of IOWT. This study demonstrated promising potential for the development of novel white tea-based products.

## 1 Introduction

Sweet osmanthus (*Osmanthus fragrans* Lour.), a member of the Oleaceae family and the genus *Osmanthus*, is characterized by slender pedicels and very short stamens. Its distribution center is primarily located in East Asia (1, 2). Sweet osmanthus flowers are edible, serve as key ingredient in various spices, as well as excellent medicinal raw materials (3). When combined with tea, they become a common ingredient for tea beverages. The distinctive aroma of sweet osmanthus makes it the primary raw material for numerous spices (4). Additionally, sweet osmanthus contains a diverse range of volatile oils, terpenoid compounds, flavonoid compounds, and other pharmacological ingredients, with a particularly high flavonoid content (5, 6). A study by Yu et al. (7) demonstrated that the total flavonoids content of sweet osmanthus flowers could reach 7.86 mg/g through ultrasonic-assisted extraction.

White tea, one of the six major tea types, is characterized by minimal fermentation and undergoes only withering and drying during its production process (8). It is renowned for its intact buds and leaves, fresh fragrance, clear yellow-green liquor, and a light, refreshing aftertaste (9). The prolonged withering process in white tea production enhances the accumulation of flavonoids, amino acids, and sugars (10). Consequently, white tea possesses the highest flavonoid content among the six major types of tea derived from the same raw leaves (11). Furthermore, the flavonoids present in white tea have been associated with a range of health benefits, including anti-mutagenic and anti-cancer effects, antibacterial and antiviral activities, antioxidative and anti-aging properties, and regulation of glycaemic levels (12, 13, 41).

Flavonoids provide numerous health benefits, but sufficient intake through our daily diet can be challenging. Consequently, individuals may supplement flavonoids through functional food products such as instant tea mixed with botanical extracts and high-flavonoid nutritional supplements (14, 15). Instant tea, a convenient and rapid beverage option, is characterized by its rapid solubility in water, eliminating the need for brewing (16). It is widely appreciated for its distinctive aroma and potential health-promoting properties. Notably, it is rich in tea polyphenols, including catechins and flavonoids, which exhibit potent antioxidant activity (17). These compounds effectively neutralize free radicals and protect cells from oxidative damage, offering powerful antioxidant and anti-inflammatory benefits that contribute to overall health (18). Research has been conducted on single-ingredient instant beverages, such as instant tea products such as instant ginger drinks, instant Oolong tea, and instant *Pu’er* tea (19–21). However, limited attention has been directed toward the formulations of instant beverages produced from blended ingredients. To our knowledge, no studies have been conducted on the development of instant sweet osmanthus white tea (IOWT). This research addresses this gap by optimizing the formulation of IOWT and investigating the impact of various processing factors on its sensory attributes, flavonoid content, and antioxidant activity. Enriching the diversity of market flavors, this study aims to broaden the scope of product innovation and applications for white tea and sweet osmanthus, providing valuable insights for future product development.

## 2 Materials and methods

### 2.1 Material and chemicals

In this study, the sweet osmanthus (*Osmanthus fragrans*) and *Shui Xian* white tea (*Camellia sinensis*) were purchased from Zhenghe Yungen White Tea Co. Ltd. (Fujian, China). The rutin standard (purity ≥98%) was acquired from Shanghai Yuanye Biotechnology Co. Ltd. (Shanghai, China). The 1,1-diphenyl-2-picrylhydrazyl and 2,2′-azinobis-3-ethylbenzthiazoline-6-sulphonate were obtained from Sigma Co. Ltd. (Shanghai, China). The *β*-cyclodextrin, erythritol, and citric acid purchased were of food grade (purchased from Henan Wanbang Chemical Technology Co. Ltd., Henan, China). Other chemicals used for analysis were analytical grade (purchased from Sinopharm Group Chemical Reagent Co. Ltd., Shanghai, China).

### 2.2 Preparation of sweet osmanthus and white tea extracts

Sweet osmanthus extract was prepared using the method described by Meng et al. (22). Sweet osmanthus was mixed with water at a 1:30 solid-to-liquid ratio (g/mL), and the mixture was subjected to ultrasonication at 120 W, 50°C for 35 min. The resulting liquor was filtered through a 200-mesh sieve to obtain the sweet osmanthus extract. Concurrently, white tea extract was prepared following the procedure outlined by Zhang et al. (23). A mixture of white tea and water at a solid-to-liquid ratio of 1:40 (g/mL) was prepared. This mixture was boiled for 5 min and subsequently strained through a 200-mesh sieve to obtain the white tea extract.

To prepare the IOWT, the sweet osmanthus and white tea extracts were mixed with varying proportions of *β*-cyclodextrin, erythritol, and citric acid. The mixtures were thoroughly stirred until all the dry ingredients were completely dissolved. Subsequently, the mixtures were centrifuged (Lishen Scientific Instrument Equipment, Neofuge-15R, Shanghai, China) at 2,795 × *g* for 10 min at −5°C. The supernatants were concentrated using a rotary evaporator (Shanghai Yarong, RE-52AA, Shanghai, China) at 45°C. Finally, concentrated extracts were obtained by spray (Huihetang Bioengineering Equipment, Bioq-8000, Shanghai, China) with a feed temperature of 150°C and a feed flow rate of 3.0 L/h to produce IOWTs (24).

### 2.3 Single-factor experimental design for the preparation of IOWT

**Figure 1:**
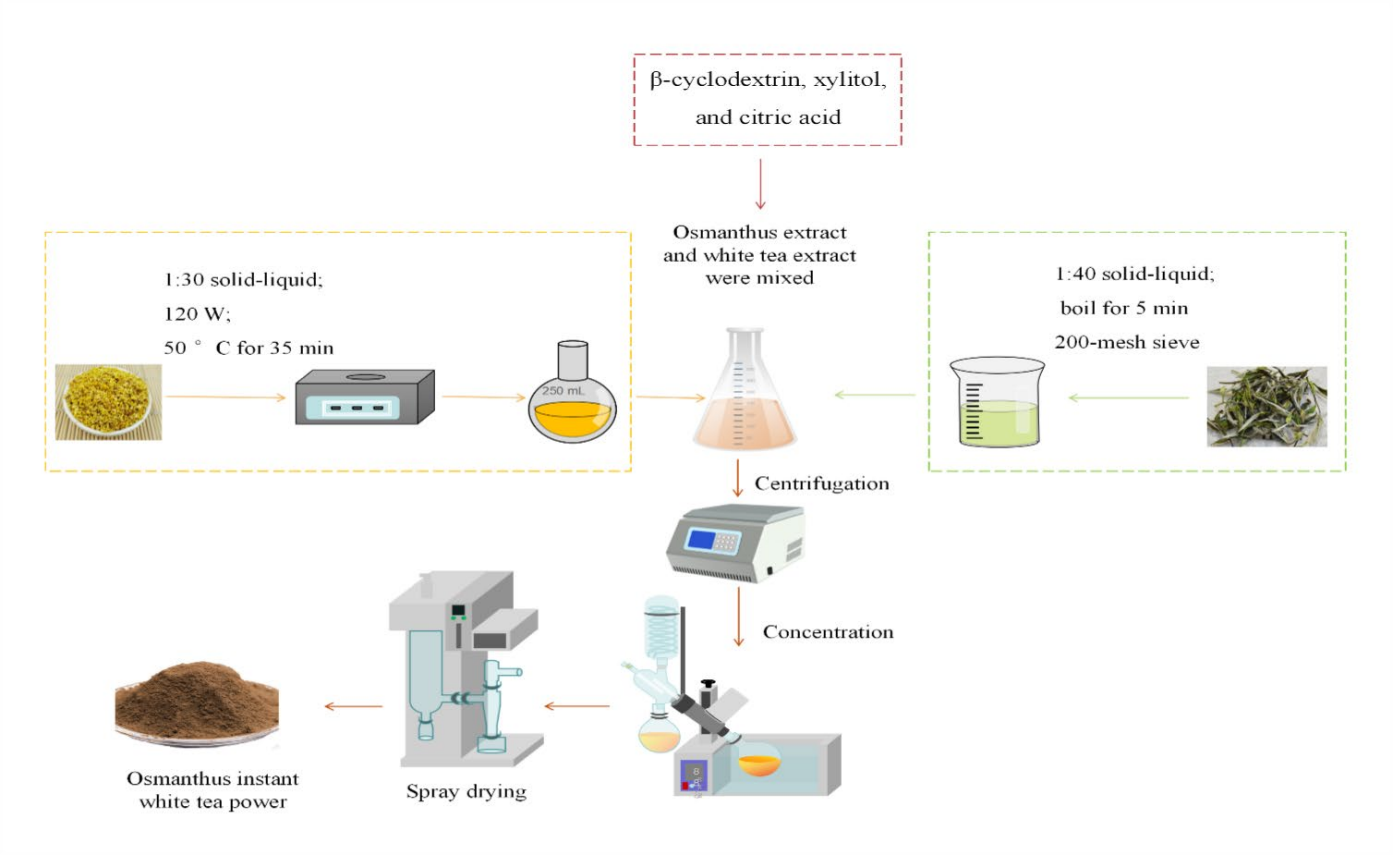
The flowchart of producing the sweet osmanthus instant white tea (IOWT) powder.

The preparation of IOWTs is divided into two distinct phases: (1) the extraction of sweet osmanthus and white tea extracts and (2) the formulation of IOWTs, as outlined in Section 2.2 (Figure 1).

#### 2.3.1 Impact of varying *β*-cyclodextrin concentrations on IOWT

Sweet osmanthus and white tea extracts were mixed in beakers at 3:10. Subsequently, 0.4% citric acid and 2% erythritol were added to the mixtures. Then, different concentrations of *β*-cyclodextrin (4, 5, 6, 7, and 8%, w/w) were added to the mixtures to investigate the impact of varying *β*-cyclodextrin concentrations on the sensory quality and total flavonoids content (TFC) of the IOWT, as outlined in Sections 2.5 and 2.6, respectively.

#### 2.3.2 Impact of varying sweet osmanthus-to-white tea ratio on IOWT

Mixtures containing 4% *β*-cyclodextrin, 0.4% citric acid, and 2% erythritol were prepared in beakers. Subsequently, sweet osmanthus and white tea extracts were added at different ratios (1:10, 2:10, 3:10, 4:10, and 5:10) to investigate the influence of these varying ratios on the sensory quality and TFC of IOWT.

#### 2.3.3 Impact of varying erythritol concentrations on IOWT

In beakers, mixtures containing sweet osmanthus and white tea in a ratio of 2:10, 4% *β*-cyclodextrin, and 0.4% citric acid were prepared. Subsequently, varying concentrations of erythritol (2, 4, 6, 8, and 10%, w/w) were added to the mixtures to evaluate the impact of different erythritol concentrations on the sensory quality and TFC of the IOWT.

#### 2.3.4 Impact of varying citric acid concentrations on IOWT

Mixtures were prepared in beakers by combining sweet osmanthus and white tea extracts in a 2:10 ratio, 6% erythritol, and 4% *β*-cyclodextrin. Subsequently, citric acid was introduced at varying concentrations (0.4, 0.6, 0.8, 1, and 1.2%, w/w) to investigate the impact of these varying concentrations of citric acid on the sensory quality and the TFC of IOWT.

### 2.4 Orthogonal test

Based on the single-factor experiments (Section 2.3), the optimal range of single factors was preliminarily screened, resulting in the selection of four factors: sweet osmanthus-to-white tea ratio, *β*-cyclodextrin concentration, erythritol concentration, and citric acid concentration. An L_9_ (3^4^) orthogonal experimental design was used with sensory scores and TFC as the evaluation indicators (Table 1).

**Table 1:** Factor levels for orthogonal test.

Level	Factor^1^			
	A	B	C	D
1	1:4	3	5	0.5
2	1:5	4	6	0.6
3	1:6	5	7	0.7

^1^A (sweet osmanthus-to-white tea ratio), B (β-cyclodextrin concentration, %), C (erythritol concentration, %), and D (citric acid concentration, %).

### 2.5 Sensory evaluation

Sensory evaluation was conducted following the methodology outlined by Lin et al. (25). The prepared IOWT was brewed with water at an optimal 1:300 (g/mL) ratio at 85°C. A panel of 10 (five males and five females) trained professional tea experts evaluated the sensory quality of the brewed IOWT. The evaluation encompassed four sensory attributes: color (maximum score of 20), aroma (maximum score of 30), taste (maximum score of 30), and solubility (maximum score of 20) (Table 2).

**Table 2:** The rating criteria for sensory evaluation of instant sweet osmanthus white tea.

Sensory evaluation	Description	Scores
Color	The liquor is clear and brightly colored	16–20
	The liquor is quite bright	11–15
	The liquor is dull and lackluster	0–10
Aroma	The unique aroma of white tea and sweet osmanthus	21–30
	A hint of sweet osmanthus fragrance	16–20
	Solely the fragrance of white tea	0–15
Taste	Rich, mellow, and well-balanced sweetness with a hint of sourness.	21–30
	Strong tea flavor, delicious	11 ~ 20
	Bitter and astringent taste, leaning toward sour or sweet	0–10
Solubility	Dissolves quickly	16–20
	Dissolves slowly	11–15
	Dissolves slowly, requires stirring	0–10

### 2.6 Determination of TFC

The TFC in the IOWT samples was assessed using the method outlined by Czechowski et al. (26). In brief, the IOWTs were brewed with water at the optimal tea-to-water (1:300, g/mL) at 85°C. Subsequently, 0.2 mL of the brewed tea samples were transferred to 10 mL test tubes, and the volume was brought up to 5 mL using 80% ethanol solution. The mixtures were thoroughly mixed before measuring the absorbance at 510 nm. The TFC (expressed in %) of the IOWT was calculated using the rutin standard curve (*y* = 10.82*x* + 0.0123, *R*^2^ = 0.997) and Equation 1.


(1) $$\begin{array}{l} \mathrm{Total flavonoids content}\;\left(\%\right)=\\ \left[\left(C\times V_{2}\times V_{3}\right)/\left(M\times V_{1}\times 10^{3}\right)\right]\times 100\% \end{array}$$

where, *C* (in mg/mL) represents the concentration of the total flavonoids, *V*_1_, *V*_2_, and *V*_3_ (in mL) represents the volume of the sample solution during measurement, the total volume of the sample solution, and the volume of the sample solution after dilution, respectively, and *M* (in g) represent the mass of the sample.

### 2.7 Antioxidant activities

The antioxidant activities of the IOWT powder at various concentrations were compared with those of Vit. C (ascorbic acid) and instant white tea. In the preparation step, 0.1 g of Vit. C, instant white tea, and IOWT powder were accurately weighed. Using distilled water, ascorbic acid, instant white tea, and IOWT solutions were prepared at varying concentrations of 0.2, 0.4, 0.6, 0.8, and 1.0 mg/mL. These sample solutions were utilized for the following *in vitro* antioxidant assays.

#### 2.7.1 DPPH radical scavenging assay

The determination of the DPPH radical scavenging activity was conducted following the method described by Chen et al. (27). In 5 mL test tubes, sample solutions (2.0 mL) were mixed with 2 mL of DPPH solution (0.3 mM, 95% ethanol) and left to react in the dark at 25°C for 30 min. For the blank group, 2 mL of the DPPH solution was mixed with 2 mL of 95% ethanol. Ethanol (95%) was used instead of DPPH as the sample reference for the reference group. Absorbances at 517 nm were measured using a UV–vis spectrophotometer (Shanghai Meipuda Instrument, UV-3200 PC, Shanghai, China). The DPPH radical scavenging activity was calculated using Equation 2:


(2) $$\begin{array}{l} \mathrm{DPPH radical scavenging activity}\;\left(\%\right)\\ =\left[1–\left(A_{0}–A_{1}\right)/A_{2}\right]\times 100\% \end{array}$$

where A_0_, A_1_ and A_2_ are the absorbance values at 517 nm of the sample, reference and blank groups, respectively.

#### 2.7.2 ABTS radical scavenging assay

The ABTS radical scavenging activity was performed according to the method described by Chen et al. (27). The ABTS solution (7 mM) and K_2_S_2_O_8_ solution (2.45 mM) were mixed equally and left in the dark at room temperature for 16 h. Subsequently, the absorbance of the mixture was adjusted to 0.70 ± 0.02 at 734 nm using phosphate buffer (5 mM, pH 7.4) before use. An equal volume of this mixture was added to the samples and then incubated in the dark. After 10 min of incubation, the absorbance of the mixtures was measured at 734 nm using the UV-3200 PC UV–vis spectrophotometer. Distilled water was used as the blank. The ABTS free radical scavenging activity was calculated using Equation 3.


(3) $$\mathrm{ABTS scavenging activity}\;\left(\%\right)=\left[\left(A_{0}-A_{1}\right)/A_{0}\right]\times 100\%\text{.}$$

where, A_0_ and A_1_ are the absorbance of the blank and sample groups, respectively.

#### 2.7.3 Hydroxyl radical scavenging assay

The activity of scavenging hydroxyl radicals was determined using the method described by Yang et al. (28). Phosphate buffer (0.5 mL, 5 M, pH 7.4) and FeSO_4_ (1.0 mL, 0.75 mM) were thoroughly mixed before 1.0 mL of H_2_O_2_ (0.1%) was added immediately and mixed. Subsequently, the mixture was added to the samples and mixed well. The mixtures were then incubated in a water bath (Changzhou Zhongjie, HH-S4, Jiangsu, China) at 37°C for 30 min. Distilled water was added to the mixtures to make the final volume 1.5 mL. Absorbances at 510 nm were measured using the UV-3200 PC UV–vis spectrophotometer. Distilled water was used as the blank. The hydroxyl radical scavenging activity was calculated using Equation 4.


(4) $$\begin{array}{l} \mathrm{Hydroxyl radical scavenging activity}\\ =\left[\left(A_{1}-A_{2}\right)/\left(A_{0}-A_{2}\right)\right]\times 100\% \end{array}$$

where, A_0_, A_1_, and A_2_ are the absorbance of the blank, negative control, and sample groups, respectively.

#### 2.7.4 Superoxide anion radical assay

The superoxide anion radical assay was performed using the method described by Yang et al. (28). Samples (2 mL) were mixed with 4.5 mL of Tris–HCl buffer (pH 8.2) containing 0.4 mL of pyrogallic acid and 1.0 mL of HCl (8.0 mM). For blank, samples were mixed with distilled water. Absorbances were measured at 325 nm using the UV-3200 PC UV–vis spectrophotometer. The superoxide anion radical scavenging activity was calculated using Equation 5.


(5) $$\begin{array}{l} \mathrm{Superoxide anion radical scavenging activity}\\ =\left[1-\left(A_{1}/A_{0}\right)\right]\times 100\% \end{array}$$

where, A_0_ and A_1_ are the absorbance of the blank and sample groups, respectively.

### 2.8 Statistical analysis

All assays were conducted in triplicate. The data is presented in mean ± standard deviation. Analysis of variance (ANOVA) and Duncan’s multiple range test were employed to analyze the data utilizing the SPSS statistical software (IBM, Chicago, United States). A *p*-value less than 0.05 was deemed statistically significant.

## 3 Results and discussion

### 3.1 Impact of *β*-cyclodextrin concentration on IOWT

**Figure 2:**
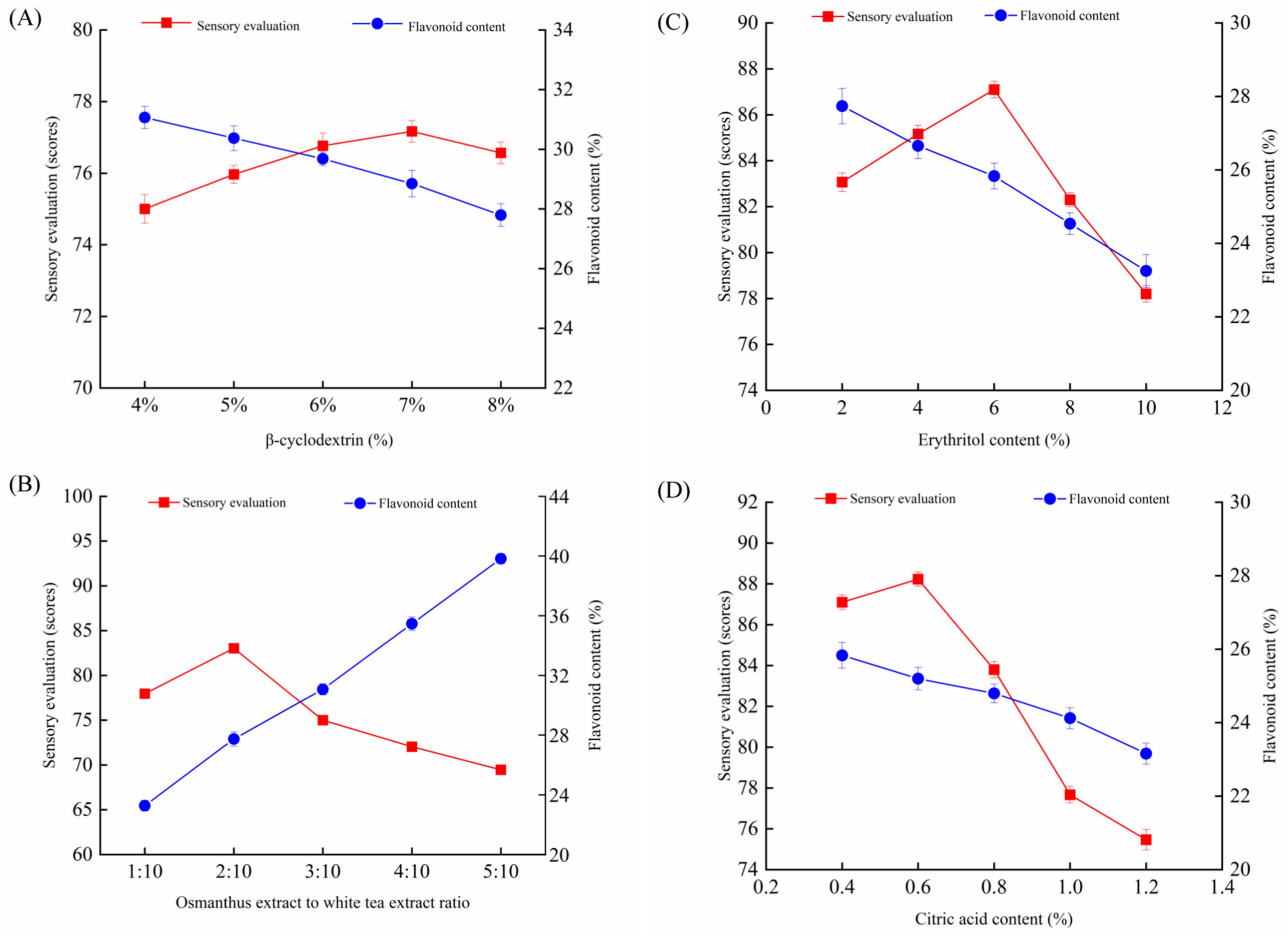
Sensory score and flavonoids content of instant sweet osmanthus white tea (IOWT) with different β-cyclodextrin concentrations **(A)**, sweet osmanthus*-*to-white tea ratio **(B)**, erythritol concentrations **(C)**, and citric acid concentrations **(D)**. Data presented as mean. Error bars represent standard deviation.

As *β*-cyclodextrin content increases from 4 to 8%, sensory evaluation scores exhibit a slight upward trend, indicating that higher *β*-cyclodextrin content enhances sensory acceptability (Figure 2A). Conversely, flavonoid content decreases consistently, suggesting a negative correlation between *β*-cyclodextrin levels and flavonoid retention. This phenomenon may be attributed to the increase in *β*-cyclodextrin content leading to the inclusion interaction between *β*-cyclodextrin and flavonoid compounds, thereby altering the physicochemical properties of flavonoids and resulting in a decline in TFC (23). Sensory scores were improved when *β*-cyclodextrin was added in concentrations ranging from 4 to 7%. This improvement can be attributed to the minimal impact of varying proportions of *β*-cyclodextrin on taste and aroma compared to its more pronounced effects on color and solubility (29). With increasing concentrations of *β*-cyclodextrin, the IOWT exhibited brighter colors and improved solubility. Since *β*-cyclodextrin possesses a sweet taste, the sensory score peaked at 77.17 with a 7% addition. Within the scope of this study, the TFC was at its highest, 31.07%, when the *β*-cyclodextrin concentration was 4%, resulting in a sensory score of 75. Considering both parameters, the influence of *β*-cyclodextrin on sensory scores was negligible. Consequently, *β*-cyclodextrin was added at 4% for subsequent single-factor experiments.

### 3.2 Impact of sweet osmanthus-to-white tea ratio on IOWT

As the sweet osmanthus-to-white tea ratio increases from 1:10 to 5:10, sensory evaluation scores peak at 2:10, followed by a sharp decline (Figure 2B). Conversely, flavonoid content rises steadily with the rising ratio, indicating a trade-off between sensory acceptability and flavonoid retention as the ratio changes. The higher the proportion of the sweet osmanthus extract, the higher the TFC and the more pronounced the sweet osmanthus fragrance. However, increasing TFC can also lead to a less desirable taste (17). The taste became bland with a 1:10 ratio, and only a faint scent of sweet osmanthus was detectable. At a 2:10 ratio, the taste became mellow and sweet, with the unique aromas of white tea and sweet osmanthus present, reaching the highest sensory score of 83.1 points and a TFC of 27.74%. At a 3:10 ratio, the tea flavor became strong and exhibited bitterness, though the sweet osmanthus fragrance was highlighted. Different solid-to-liquid ratios had a notable impact on the sensory attributes of the final product, including taste, color, and concentration (30). Considering all factors, the sweet osmanthus-to-white tea ratio of 2:10 (or 1:5) offers the best balance and was used for subsequent single-factor experiments.

### 3.3 Impact of erythritol concentration on IOWT

As erythritol content increases from 2 to 10%, sensory evaluation scores peak at ~6%, followed by a rapid decline (Figure 2C). This observation suggested that moderate levels of erythritol are optimal for sensory acceptability, while higher levels diminish consumer preference. Similarly, flavonoid content decreases gradually with increasing erythritol content, indicating a negative impact on flavonoid retention at higher erythritol concentrations. Consistent with previous findings, Kamkaew et al. (31) demonstrated that sweeteners significantly influence the stability of flavonoid solutions. In their study, Wojtyś et al. (32) investigated the osmotic dehydration of Japanese quince fruit in erythritol solution. Their findings revealed a notable impact of erythritol on the flavonoid content of the fruit, suggesting that sweeteners may mask the bitterness of flavonoids. The product lacks sufficient sweetness at a concentration of 2%, resulting in a sensory score of 83.1 points. At a concentration of 6%, the product exhibits a rich tea flavor that is sweet and sour, making it delectable, with the highest sensory score of 87.1 points and a TFC of 25.83%. The product’s sweetness became excessive at a concentration of 10%, leading to the lowest sensory scores and TFC. Considering all factors, the optimal taste is achieved with 6% erythritol. This concentration was used for subsequent single-factor experiments.

### 3.4 Impact of citric acid concentration on IOWT

As the citric acid content increases from 0.4 to 1.2%, the sensory evaluation scores peak at 0.6% and decline sharply, indicating that higher citric acid concentrations adversely affect sensory acceptability (Figure 2D). Conversely, flavonoid content progressively decreases with increasing citric acid content, suggesting a potential degradation of flavonoids at elevated acidity levels. The study by Santosh et al. (33) proposed that the reduction in flavonoid content may be attributed to multiple H^+^ ions in citric acid. At a citric acid concentration of 0.6%, the sensory score was 88.2 points (the highest), devoid of any sour taste, and the TFC was 25.20%. At a citric acid concentration of 1.0%, the taste began to exhibit sourness, and at 1.2% citric acid, the taste became notably sour and astringent, with the lowest TFC. The outcomes were comparable to those reported in a recent study conducted by Wang et al. (34). The study demonstrated that reducing citric acid levels alleviated sourness and astringency, enhancing the mouthfeel and releasing total flavonoid content. Notably, the antioxidant activity exhibited a notable increase. Considering all factors, the optimal taste is achieved with a citric acid concentration of 0.6%. This concentration was used for subsequent single-factor experiments.

### 3.5 Orthogonal experimental results and analysis

From the sensory range values (R_I_), it is evident that the factors influencing the sensory scores of the product were ranked in the order of A > C > D > B (Table 3). This indicates that the factors affecting the product’s sensory scores are the sweet osmanthus-to-white tea ratio (A), erythritol concentration (C), citric acid concentration (D), and *β*-cyclodextrin concentration (B), respectively. According to the flavonoid range analysis (R_II_) results, the primary factors affecting the flavonoid content of the product were ranked as A > C > B > D, which translates to the sweet osmanthus-to-white tea ratio > erythritol > *β*-cyclodextrin > citric acid. Among these, the sweet osmanthus-to-white tea ratio has the most significant impact on the IOWT’s sensory scores and the TFC. A study by Jiang et al. (19) demonstrated that various experimental factors had varying effects on the process optimization, physicochemical properties, and antioxidant activity of instant black tea.

**Table 3:** Orthogonal experimental design and results analysis.

Number of experiments	Factor^1^				Sensory score	Flavonoid content (%)
	A	B	C	D		
1	1	1	1	1	77.5	29.42
2	1	2	2	2	80.2	28.63
3	1	3	3	3	78.0	27.33
4	2	1	2	3	86.1	26.51
5	2	2	3	1	88.7	25.31
6	2	3	1	2	85.0	25.95
7	3	1	3	2	82.4	23.51
8	3	2	1	3	80.6	24.35
9	3	3	2	1	84.3	23.54
K_I1_	78.567	82.000	81.033	83.500		
K_I2_	86.600	83.167	83.533	82.533		
K_I3_	82.433	82.433	83.033	81.567		
R_I_	8.033	1.167	2.500	1.933		
K_II1_	28.460	26.480	26.573	26.090		
K_II2_	25.923	26.097	26.227	26.030		
K_II3_	23.800	25.607	25.383	26.063		
R_II_	4.660	0.873	1.190	0.027		
Optimal Level	A_2_	B_2_	C_2_	D_1_		

^1^A (sweet osmanthus-to-white tea ratio), B (β-cyclodextrin concentration, %), C (erythritol concentration, %), and D (citric acid concentration, %).

### 3.6 Validation experiment

The combination A_2_B_2_C_2_D_1_ was the optimal formulation for the IOWT (Table 3), which contradicts the highest-scoring experiment number 5 in the orthogonal test. Consequently, this formulation was retested for validation. The results showed that the sensory score and TFC of A_2_B_2_C_2_D_1_ were slightly higher than those of A_2_B_2_C_3_D_1_, with a sensory score of 89.5 (characterized by a unique aroma of white tea and sweet osmanthus fragrant, a rich and sweet-tasting, a clear and bright color, and a rapid dissolution rate). Furthermore, the TFC of A_2_B_2_C_2_D_1_ was 25.76%. Therefore, the optimal IOWT formulation was determined to be A_2_B_2_C_2_D_1_, which corresponds to a sweet osmanthus-to-white tea ratio of 1:5, 4% *β*-cyclodextrin, 6% erythritol, and 0.5% citric acid. Recently, Jiang et al. (19) employed sequential inoculation with golden flower (*Eurotium cristatum*) and *Aspergillus niger* in liquid-state fermentation to produce instant black tea and similarly conducted validation experiments to investigate the influencing factors.

### 3.7 Comparison of the flavonoid content

Compared to the TFC in the white tea extract and IOWT, the TFC was found to have increased from 6.75 to 25.76%, a rise of 3.82 times (Table 4). Simultaneously, compared to the TFC of instant white tea, the TFC of IOWT was 36.30% higher. This increase in the TFC indicated that after adding sweet osmanthus extract under the same processing conditions, there was a significant increase in the TFC of the instant white tea, eliminating the possibility of factors related to the processing technique.

**Table 4:** Comparison in flavonoid content among different samples.

Samples	White tea extract	Sweet osmanthus white tea liquor	Instant white tea	Instant sweet osmanthus white tea
Flavonoid content (%)	6.75	8.80	18.90	25.76

Chen et al. (41) investigated ultrasonic-assisted extraction of total flavonoids in sweet osmanthus, resulting in an extraction rate of 17.7%. Hu et al. (42) analyzed white tea’s physical and chemical composition and reported a flavonoid content ranging from 0.51 to 1.43%. Wang et al. (35), Zhou et al. (36), and Ning et al. (43) also analyzed the flavonoid components in sweet osmanthus and white tea, identifying several similarities, including quercetin, catechins, and rutin. However, the specific concentrations and other components vary between the two. Sweet osmanthus contains a higher variety of flavonoid glycosides and hesperidin, while white tea primarily comprises catechin compounds, particularly epicatechin gallate (EGCG). Consequently, combining the flavonoids from white tea and sweet osmanthus can substantially enhance the antioxidant effect through complementary species and synergistic scavenging of free radicals.

### 3.8 Antioxidant activities of IOWT

#### 3.8.1 Scavenging effects of IOWT on DPPH radicals

**Figure 3:**
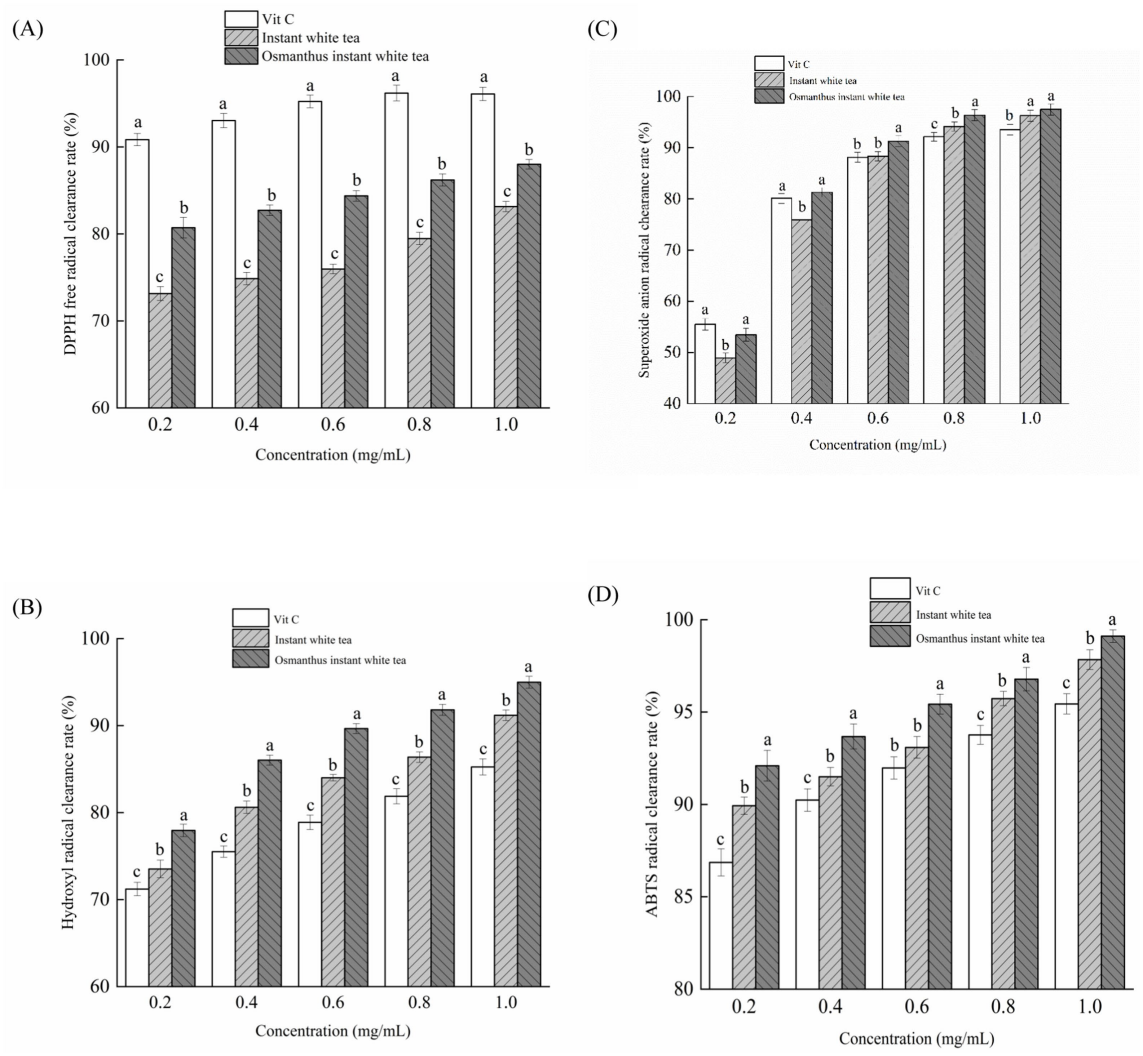
Scavenging rates of DPPH free radical **(A)**, hydroxyl radical **(B)**, superoxide anion radical **(C)**, and ABTS radical **(D)** of samples with different mass concentrations. Data are presented as mean (*n* = 3). Error bars represent standard deviation. Bars with different lowercase letters within the same concentration indicate significant (*p <* 0.05) differences between groups.

Within the concentration range of 0.2–1.0 mg/mL, the clearance rates of the DPPH free radicals by the various samples were found to increase with the increase in concentration (Figure 3A). When the concentration reached 1.0 mg/mL, the clearance rate of instant white tea was observed to be 83.14%. At the same time, that of IOWT was 88%. The DPPH radical scavenging activity of instant white tea and IOWT exhibited a dose-dependent increase with rising concentrations. However, their scavenging efficiencies remained lower than that of Vit. C, which achieved a clearance rate of 96.09%. This result is consistent with the findings of Zhao et al. (37), who demonstrated that the clearance rate of DPPH free radicals by chinaroot extract (*Smilax glabra* Roxb.) was comparable to ascorbic acid, indicating substantial antioxidant activities. At the same concentration, the clearance rates of DPPH free radicals were in the following order: Vit. C > IOWT > instant white tea.

#### 3.8.2 Scavenging effects of IOWT on hydroxyl free radicals

It was observed that with the increase in concentration, the clearance rates of hydroxyl free radicals by the various samples gradually increased, exhibiting an upward trend (Figure 3B). At a concentration of 0.2 mg/mL, the clearance rates of hydroxyl free radicals by Vit. C, instant white tea, and IOWT were observed to be 71.21, 73.52, and 77.96%, respectively. When the concentration reached 1.0 mg/mL, these rates increased to 85.25, 91.19, and 94.99%, respectively. At a concentration of 1.0 mg/mL, the clearance rates of hydroxyl free radicals by instant white tea and IOWT were higher than that of Vit. C by 6.97 and 11.43%, respectively. At the same concentration, the samples’ clearance rates of hydroxyl free radicals were in the order of IOWT > instant white tea > vit. C. The study by Wang et al. (35) demonstrated that the flavonoids from goji berry (*Lycium barbarum*) exhibited a strong scavenging rate for hydroxyl radicals, comparable to ascorbic acid, and exhibited notable antioxidant capabilities.

#### 3.8.3 Scavenging effects of IOWT on superoxide anion radicals

Upon increasing the concentration from 0.2 to 0.4 mg/mL, the efficacy of various samples in rapidly eliminating superoxide radicals was notably enhanced (Figure 3C). The clearance rates of superoxide anion radicals by Vit. C, instant white tea, and IOWT increased from 55.47, 48.93, and 53.44% to 80.09, 75.91, and 81.29%, respectively. Notably, when the concentration reached 0.6 mg/mL, the ability of IOWT to eliminate superoxide anion radicals significantly (*p* < 0.05) surpassed that of Vit. C. Furthermore, when the concentration exceeded 0.8 mg/mL, both instant white tea and IOWT exhibited a more significant (*p* < 0.05) scavenging capacity for superoxide anion radicals than Vit. C. A recent study by Yang et al. (28) revealed that flavonoids exhibited a more substantial scavenging rate for superoxide anion radicals than ascorbic acid, underscoring their significant antioxidant activity.

#### 3.8.4 Scavenging effects of IOWT on ABTS radicals

Within the concentration range of 0.2–1.0 mg/mL, the capability of instant white tea and IOWT to eliminate ABTS radicals was observed to be superior to that of Vit. C (Figure 3D). As the concentration increased, the clearance effects of Vit. C, instant white tea, and IOWT on ABTS radicals exhibited a dose-dependent growth pattern. At a concentration of 1.0 mg/mL, the abilities of instant white tea and IOWT to eliminate ABTS radicals reached 97.83 and 99.11%, respectively, surpassing those of Vit. C by 2.5 and 3.85%, respectively. White tea flavonoids primarily consist of catechin compounds, particularly EGCG, which exhibit substantial antioxidant properties by scavenging reactive oxygen species (ROS), such as superoxide anions and hydroxyl radicals (25, 36). This protective role is attributed to the flavonoids’ ability to neutralize oxidative stress. Additionally, the flavonoids of osmanthus fragrans are predominantly quercetin, rutin, and a diverse range of flavonoid glycosides (5). Notably, these glycosides exhibit metal-chelating properties, thereby enhancing the activity of antioxidant enzymes and modulating oxidative stress signaling pathways (38). A study conducted by (39) revealed that the ethanol extracts of winter daffodil (*Sternbergia lutea* ssp. *sicula*) exhibited the strongest ABTS scavenging activity with the IC_50_ value of 0.1 mg/mL.

## 4 Conclusion

This study successfully optimized the formulation of IOWT using an orthogonal experimental design, resulting in a blend of sweet osmanthus-to-white tea ratio of 1:5, 4% *β*-cyclodextrin, 6% erythritol, and 0.5% citric acid. The optimized IOWT exhibited high sensory acceptability and significant antioxidant activity, as evidenced by its superior scavenging effects on DPPH, hydroxyl, superoxide anion, and ABTS radicals compared to instant white tea and ascorbic acid. The elevated TFC in IOWT underscores the synergistic effect of sweet osmanthus and white tea, suggesting its potential as a functional beverage with enhanced health benefits. This study primarily focused on *in vitro* antioxidant activity, and *in vivo* studies are necessary to validate these findings and explore bioavailability and long-term health impacts. Furthermore, future directions on identifying volatile and flavonoid compounds and the stability of these compounds during storage will further substantiate the potential of IOWT as a health-promoting functional beverage and support its commercial application.

## Data Availability

The original contributions presented in the study are included in the article/supplementary material, further inquiries can be directed to the corresponding authors.

## References

[1] Fu CC Xu FY Qian YC Koo HL Duan YF Weng GM. Secondary metabolites of *Osmanthus fragrans*: metabolism and medicinal value. Front Pharmacol. (2022) 13:922204. doi: 10.3389/fphar.2022.922204, PMID: 35924042 PMC9340074

[2] Wang B Luan F Bao Y Peng X Rao Z Tang Q. Traditional uses, phytochemical constituents and pharmacological properties of *Osmanthus fragrans*: a review. J Ethnopharmacol. (2022) 293:115273. doi: 10.1016/j.jep.2022.115273, PMID: 35405258

[3] Sheng X Lin Y Cao J Ning Y Pang X Wu J. Comparative evaluation of key aroma-active compounds in sweet *osmanthus* (*Osmanthus fragrans* Lour.) with different enzymatic treatments. J Agric Food Chem. (2021) 69:332–44. doi: 10.1021/acs.jafc.0c06244, PMID: 33370113

[4] Xiong YC Luk J Cheong MW Curran P Liu SQ Ng KH. Biotransformation of volatiles in fermented Osmanthus (*Osmanthus fragrans*) flowers by yeast. J Essent Oil-Bear Plants. (2017) 20:298–313. doi: 10.1080/0972060x.2017.1319298

[5] Wu LP Liu JY Huang WS Wang YX Chen Q Lu BY. Exploration of *Osmanthus fragrans* Lour.'s composition, nutraceutical functions and applications. Food Chem. (2022) 377:131853. doi: 10.1016/j.foodchem.2021.131853 34990948

[6] Yang J Gu T Lu Y Xu Y Gan RY Ng SB. Edible *Osmanthus fragrans* flowers: aroma and functional components, beneficial functions, and applications. Crit Rev Food Sci Nutr. (2023) 64:10055–68. doi: 10.1080/10408398.2023.2220130, PMID: 37287270

[7] Yu J Lou Q Zheng X Cui Z Fu J. Sequential combination of microwave- and ultrasound-assisted extraction of total flavonoids from *Osmanthus fragrans* Lour. Flowers. Molecules. (2017) 22:2216. doi: 10.3390/molecules22122216, PMID: 29236089 PMC6149695

[8] Wang P Zhao B Yin Z Gao X Liu M. Structure elucidation and anticancer activity of a heteropolysaccharide from white tea. Carbohydr Polym. (2024) 333:121976. doi: 10.1016/j.carbpol.2024.121976, PMID: 38494228

[9] Hao Z Feng J Chen Q Lin H Zhou X Zhuang J. Comparative volatiles profiling in milk-flavored white tea and traditional white tea Shoumei via HS-SPME-GC-TOFMS and OAV analyses. Food Chem X. (2023) 18:100710. doi: 10.1016/j.fochx.2023.100710, PMID: 37397202 PMC10314143

[10] Tan J Engelhardt UH Lin Z Kaiser N Maiwald B. Flavonoids, phenolic acids, alkaloids and theanine in different types of authentic Chinese white tea samples. J Food Compost Anal. (2017) 57:8–15. doi: 10.1016/j.jfca.2016.12.011

[11] Zhao CN Tang GY Cao SY Xu XY Gan RY Liu Q. Phenolic profiles and antioxidant activities of 30 tea infusions from green, black, oolong, white, yellow and dark teas. Antioxidants. (2019) 8:215. doi: 10.3390/antiox8070215, PMID: 31295859 PMC6680489

[12] Li C He J Yang Y Gou Y Wang Z Chen H. White tip silver needle (slightly fermented white tea) flavonoids help prevent aging via antioxidative and anti-inflammatory effects. Drug Des Devel Ther. (2021) 15:1441–57. doi: 10.2147/DDDT.S304885, PMID: 33833503 PMC8020812

[13] Sanlier N Atik İ Atik A. A minireview of effects of white tea consumption on diseases. Trends Food Sci Tech. (2018) 82:82–8. doi: 10.1016/j.tifs.2018.10.004

[14] Pei R Liu X Bolling B. Flavonoids and gut health. Curr Opin Biotechnol. (2020) 61:153–9. doi: 10.1016/j.copbio.2019.12.018, PMID: 31954357

[15] Tao H Li L He Y Zhang X Zhao Y Wang Q. Flavonoids in vegetables: improvement of dietary flavonoids by metabolic engineering to promote health. Crit Rev Food Sci Nutr. (2024) 64:3220–34. doi: 10.1080/10408398.2022.2131726, PMID: 36218329

[16] Liang S Granato D Zou C Gao Y Zhu Y Zhang L. Processing technologies for manufacturing tea beverages: from traditional to advanced hybrid processes. Trends Food Sci Tech. (2021) 118:431–46. doi: 10.1016/j.tifs.2021.10.016

[17] Liu HY Liu Y Mai YH Guo H He XQ Xia Y. Phenolic content, main flavonoids, and antioxidant capacity of instant sweet tea (*Lithocarpus litseifolius* [Hance] Chun) prepared with different raw materials and drying methods. Food Secur. (2021) 10:1930. doi: 10.3390/foods10081930, PMID: 34441707 PMC8394704

[18] Pastoriza S Mesias M Cabrera C Rufian-Henares JA. Healthy properties of green and white teas: an update. Food Funct. (2017) 8:2650–62. doi: 10.1039/c7fo00611j, PMID: 28640307

[19] Jiang Q-x Li L-j Chen F Rong B Ni H Zheng F-p. β-Glucosidase improve the aroma of the tea infusion made from a spray-dried oolong tea instant. LWT. (2022) 159:113175:113175. doi: 10.1016/j.lwt.2022.113175, PMID: 39822849

[20] Nhan NPT Khang VC Van Hung P Thi Tuu T Anh NHT Nhan LTH. Study of hydrolysis and production of instant ginger (*Zingiber officinale*) tea. Open Chem. (2023) 21:20230363. doi: 10.1515/chem-2023-0363

[21] Zhang T Ni H Qiu XJ Li T Zhang LZ Li LJ. Suppressive interaction approach for masking stale note of instant ripened Pu-erh tea products. Molecules. (2019) 24:4473. doi: 10.3390/molecules24244473, PMID: 31817626 PMC6943613

[22] Meng X Wang JQ Wang F Gao Y Fu CH Du Q. Moisture content of tea dhool for the scenting process affects the aroma quality and volatile compounds of *osmanthus* black tea. Food Chem. (2024) 438:138051. doi: 10.1016/j.foodchem.2023.138051, PMID: 38056097

[23] Zhang Y-H Chen G-S Chen J-X Liu Z-Q Yu L-Y Yin J-F. Effects of β-cyclodextrin and sodium ascorbate on the chemical compositions and sensory quality of instant green tea powder during storage. J Chem. (2019) 2019:1–7. doi: 10.1155/2019/5618723

[24] Wang C Li J Zhang Y He Z Zhang Y Zhang X. Effects of electrostatic spray drying on the sensory qualities, aroma profile and microstructural features of instant Puerh tea. Food Chem. (2022) 373:131546. doi: 10.1016/j.foodchem.2021.131546, PMID: 34799127

[25] Lin YP Huang YB Zhou S Li XL Tao YK Pan YN. A newly-discovered tea population variety processed Bai mu Dan white tea: flavor characteristics and chemical basis. Food Chem. (2024) 446:138851. doi: 10.1016/j.foodchem.2024.138851, PMID: 38428080

[26] Czechowski T Rinaldi MA Famodimu MT Van Veelen M Larson TR Winzer T. Flavonoid versus artemisinin anti-malarial activity in *Artemisia annua* whole-leaf extracts. Front Plant Sci. (2019) 10:984. doi: 10.3389/fpls.2019.00984, PMID: 31417596 PMC6683762

[27] Chen S Yang Q Chen X Tian Y Liu Z Wang S. Bioactive peptides derived from crimson snapper and in vivo anti-aging effects on fat diet-induced high fat *Drosophila melanogaster*. Food Funct. (2020) 11:524–33. doi: 10.1039/c9fo01414d, PMID: 31844865

[28] Yang T Hu Y Yan Y Zhou W Chen G Zeng X. Characterization and evaluation of antioxidant and anti-inflammatory activities of flavonoids from the fruits of *Lycium barbarum*. Food Secur. (2022) 11:306. doi: 10.3390/foods11030306, PMID: 35159457 PMC8834156

[29] Dai Q Liu S Jin H Jiang Y Xia T. Effects of additive β-cyclodextrin on the performances of green tea infusion. J Chem. (2019) 2019:1–7. doi: 10.1155/2019/7428514, PMID: 38550227

[30] Sayuti N Kamarudin A Saad N Ab-Razak N Mohd Esa N. Optimized green extraction conditions of matcha green tea (*Camellia sinensis*) using central composite design for maximal polyphenol and antioxidant contents. Bioresources. (2021) 16:3255–71. doi: 10.15376/biores.16.2.3255-3271

[31] Kamkaew N Paracha TU Ingkaninan K Waranuch N Chootip K. Vasodilatory effects and mechanisms of action of *Bacopa monnieri* active compounds on rat mesenteric arteries. Molecules. (2019) 24:2243. doi: 10.3390/molecules24122243, PMID: 31208086 PMC6630913

[32] Wojtyś A Pietrzyk S Bogacz S Witkowicz R. Osmotic dehydration of Japanese quince (*Chaenomeles japonica*) fruits in erythritol solutions: impact of processing conditions on the kinetic parameters and on physicochemical and antioxidant properties of the fruits. Molecules. (2024) 29:5524. doi: 10.3390/molecules29235524, PMID: 39683684 PMC11643805

[33] Santosh KC Liu M Zhang Q Fan K Shi Y Ruan J. Metabolic changes of amino acids and flavonoids in tea plants in response to inorganic phosphate limitation. Int J Mol Sci. (2018) 19:3683. doi: 10.3390/ijms19113683, PMID: 30469347 PMC6274676

[34] Wang C Wang Y Shan WT Han YQ Li X. Solid-state fermentation with lactic acid bacteria of citric acid degrading for hawthorn fruit improvement: sensory, flavor, antioxidant properties. Food Biosci. (2024) 62:105402. doi: 10.1016/j.fbio.2024.105402

[35] Wang Y Fu J Zhang C Zhao H. HPLC-DAD-ESI-MS analysis of flavonoids from leaves of different cultivars of sweet osmanthus. Molecules. (2016) 21:1224. doi: 10.3390/molecules21091224, PMID: 27649119 PMC6274377

[36] Zhou S Zhang J Ma S Ou C Feng X Pan Y. Recent advances on white tea: manufacturing, compositions, aging characteristics and bioactivities. Trends Food Sci Technol. (2023) 134:41–55. doi: 10.1016/j.tifs.2023.02.016

[37] Zhao X Chen R Shi Y Zhang X Tian C Xia D. Antioxidant and anti-inflammatory activities of six flavonoids from *Smilax glabra* Roxb. Molecules. (2020) 25:5295. doi: 10.3390/molecules25225295, PMID: 33202848 PMC7697956

[38] Wang Q Gao G Chen X Liu X Dong B Wang Y. Genetic studies on continuous flowering in woody plant *Osmanthus fragrans*. Front Plant Sci. (2022) 13:1049479. doi: 10.3389/fpls.2022.1049479, PMID: 36407607 PMC9671776

[39] Can Ağca A Yazgan Ekici AN Yılmaz Sarıaltın S Çoban T Saltan İşcan G Sever Yılmaz B. Antioxidant, anti-inflammatory and antidiabetic activity of two *Sternbergia* taxons from Turkey. S Afr J Bot. (2021) 136:105–9. doi: 10.1016/j.sajb.2020.04.002

[40] Hinojosa-Nogueira D Perez-Burillo S Pastoriza de la Cueva S Rufian-Henares JA. Green and white teas as health-promoting foods. Food Funct. (2021) 12:3799–819. doi: 10.1039/d1fo00261a, PMID: 33977999

[41] Chen PZ Liu JS Liu RL Ge XB. Study on ultrasonic-assisted extraction of the total flavonoids in Osmanthus fragrans var. Aurantiacus and its antioxidative activity. Food Res Dev. (2015) 36:47–51. doi: 10.3969/j.issn.1005-6521.2015.23.012

[42] Hu JX. Analysis of physical and chemical components of white tea and structural identification of anthocyanin. Master’s thesis. Zhejiang University. (2020). doi: 10.27461/d.cnki.gzjdx.2020.001151

[43] Ning JM Wan DD Song YS Zhang ZZ Luo XJ. Chemical constituents analysis of white tea of different qualities and different storage times. Eur Food Res Technol. (2016) 242:2093–104. doi: 10.1007/s00217-016-2706-0

